# Supplementary material for: COVID-19 and regional differences in the timeliness of hip-fracture surgery: an interrupted time-series analysis
Source: PeerJ. 2021 Aug 31;9:e12046. doi: 10.7717/peerj.12046 (PMC8415287; doi:10.7717/peerj.12046)
Supplement: Supplemental Information 1 [file peerj-09-12046-s001.docx]

**SUPPLEMENTS**

**Fig. S1** Prevalence of COVID-19-associated hospitalizations and cumulative incidence of COVID-19 cases (×100,000 population) in Piedmont, Emilia-Romagna and Italy between February 24, 2020 and June 9, 2020.

*Note:* Dashed vertical line is Italy’s COVID-19 national lockdown (March 10, 2020).

*Source:* *Dipartimento della protezione civile*.

**Table S1** Characteristics of hip-fracture patients and admitting hospitals in Piedmont and Emilia-Romagna between November 20, 2019 and June 30, 2020, by 13-week period; values are counts (percentages).

| Hospital characteristic | Piedmont | | Emilia-Romagna | | |  |
| --- | --- | --- | --- | --- | --- | --- |
|  | Nov-20 to | Mar-11 to | | Nov-20 to | Mar-11 to | |
|  | Mar-10 | June-30 | | Mar-10 | June-30 | |
| Sex |  |  | |  |  | |
| Male | 413 (24.3) | 357 (25.7) | | 444 (25.7) | 372 (25.4) | |
| Female | 1285 (75.7) | 1031 (74.3) | | 1286 (74.3) | 1093 (74.6) | |
| Age group, y |  |  | |  |  | |
| <80 | 427 (25.1) | 352 (25.4) | | 449 (26.0) | 360 (24.6) | |
| 80–84 | 393 (23.1) | 337 (24.3) | | 385 (22.3) | 333 (22.7) | |
| 85–89 | 491 (28.9) | 401 (28.9) | | 458 (26.5) | 403 (27.5) | |
| ≥90 | 387 (22.8) | 298 (21.5) | | 438 (25.3) | 369 (25.2) | |
| Charlson index score |  |  | |  |  | |
| 0 | 1442 (84.9) | 1176 (84.7) | | 1465 (84.7) | 1277 (87.2) | |
| 1 | 199 (11.7) | 157 (11.3) | | 172 (9.9) | 122 (8.3) | |
| ≥2 | 57 (3.4) | 55 (4.0) | | 93 (5.4) | 66 (4.5) | |
| Status/ownership |  |  | |  |  | |
| Local healthcare authority | 1255 (73.9) | 935 (67.4) | | 1250 (72.3) | 958 (65.4) | |
| Research/teaching | 407 (24.0) | 436 (31.4) | | 473 (27.3) | 502 (34.3) | |
| Private | 36 (2.1) | 17 (1.2) | | 7 (0.4) | 5 (0.3) | |
| Location |  |  | |  |  | |
| Cities | 684 (40.3) | 680 (49.0) | | 1211 (70.0) | 1048 (71.5) | |
| Towns/suburbs | 1014 (59.7) | 708 (51.0) | | 455 (26.3) | 371 (25.3) | |
| Rural areas | 0 (0.0) | 0 (0.0) | | 64 (3.7) | 46 (3.1) | |
| Avg. annual n. of hip fractures |  |  | |  |  | |
| <50 | 5 (0.3) | 5 (0.4) | | 70 (4.0) | 30 (2.0) | |
| 50–149 | 234 (13.8) | 157 (11.3) | | 209 (12.1) | 164 (11.2) | |
| 150–249 | 621 (36.6) | 425 (30.6) | | 286 (16.5) | 367 (25.1) | |
| ≥250 | 838 (49.4) | 801 (57.7) | | 1165 (67.3) | 904 (61.7) | |

**Table S2** Hip-fracture surgery (%) initiated within 2 days, within 1 day and on the same day as hospital admission in Piedmont and Emilia-Romagna, Italy, by 13-week observation period; secondary diagnoses of upper femur fracture are excluded (153 in Piedmont and 125 in Emilia-Romagna).

| Thirteen-week observation period | Piedmont | | | | | | Emilia-Romagna | | | | | |
| --- | --- | --- | --- | --- | --- | --- | --- | --- | --- | --- | --- | --- |
|  | Crude rate | | | Standardized rate ^a^ | | | Crude rate | | | Standardized rate ^a^ | | |
|  | Day 2 | Day 1 | Day 0 | Day 2 | Day 1 | Day 0 | Day 2 | Day 1 | Day 0 | Day 2 | Day 1 | Day 0 |
| Dec-11-2018 to Mar-11-2019 | 72.3 | 46.7 | 13.4 | 72.0 | 46.4 | 13.4 | 79.2 | 43.1 | 8.8 | 79.1 | 43.0 | 8.6 |
| Mar-12-2019 to Jun-10-2019 | 70.9 | 44.6 | 14.0 | 70.9 | 44.7 | 14.0 | 77.1 | 44.4 | 8.9 | 77.4 | 44.7 | 9.1 |
| Dec-11-2019 to Mar-10-2020 | 71.2 | 46.3 | 12.9 | 70.9 | 46.1 | 12.8 | 80.6 | 47.8 | 10.4 | 80.4 | 47.8 | 10.5 |
| Mar-11-2020 to Jun-09-2020 | 71.3 | 47.8 | 12.6 | 71.3 | 47.8 | 12.6 | 71.1 | 43.1 | 7.7 | 71.0 | 43.0 | 7.6 |

^a^ By sex, age and enhanced Charlson index with direct standardization to the overall composition of hip fractures included in the study.

**Fig. S2** Interrupted time-series analysis of weekly sex-, age- and comorbidity-standardized percentages of hip-fracture surgery in Piedmont and Emilia-Romagna in the 13 weeks before and after Italy’s COVID-19 national lockdown (dashed vertical line); secondary diagnoses of upper femur fracture are excluded (153 in Piedmont and 125 in Emilia-Romagna).

*Note:* Data observed the year before (2018/19) are used for comparison. The last day of the control period is June 10, 2019, because 2019 is a common (non-leap) year.
